# Essential Oil Yield Pattern and Antibacterial and Insecticidal Activities of* Trachyspermum ammi* and* Myristica fragrans*

**DOI:** 10.1155/2016/1428194

**Published:** 2016-04-12

**Authors:** Rajgovind Soni, Gaurav Sharma, Nakuleshwar Dut Jasuja

**Affiliations:** School of Sciences, Suresh Gyan Vihar University, Jaipur, Rajasthan 302017, India

## Abstract

Two Indian spices,* Trachyspermum ammi *and* Myristica fragrans, *were studied for their essential oil (EO) yielding pattern, insecticidal activity, antibacterial activity, and composition. The essential oils (EOs) of* T. ammi *(1.94 ± 30 mL/100 gm) and* M. fragrans *(5.93 ± 90 mL/100 gm) were extracted using hydrodistillation method. In Gas Chromatography analysis, the beta-pinene, alpha-pinene, alpha-p-menth-1-en-4-ol, Limonene, and elemicin were found as major constituents of* T. ammi *essential oil whereas* M. fragrans *essential oil mostly contains Gamma-Terpinolene, p-Cymene, Thymol, and beta-pinene. The insecticidal activities of EO were demonstrated using LC_50_ values against* Plodia interpunctella *and EO of* T. ammi *was found comparatively more effective than EO of* M. fragrans. *Further, individual EO and combination of essential oil were examined for antibacterial activity against three Gram (−) bacterial strains (*E. coli*-MTCC 443,* P. vulgaris*-MTCC 1771, and* K. pneumoniae*-MTCC number 7028) and three Gram (+) bacterial strains (*S. aureus*-MTCC 3381,* B. subtilis*-MTCC 10619, and* B. megaterium*-MTCC 2412) by well agar diffusion method. The essential oil in combination (CEO) exhibited higher antibacterial activity as compared with individual essential oils.

## 1. Introduction

Much of human nutrition depends on plants, either directly or indirectly. In the ancient time only plants, animals, rocks, and trees are the pharmaceutical giants for men. Nowadays essential oils are being used for healing in holistic manner and, optimistically, their medicinally active constituents may add their contribution to the treatment of various ailments [1–3]. These oils contain about one hundred to two hundred different carbon and hydrogen based compounds called terpenes or hydrocarbons [4]. The alcohols and phenolic compounds, for example, terpenes, are mainly responsible [5, 6] for antibacterial, antifungal, antiviral, and insecticidal activities of essential oil against many microorganisms [7–11]. Impudently, few species of insects [12] are serious threat for public and may cause harm to human, farm animals, and crops; therefore, EO may be used in formulation of insecticidal application. In vitro antimicrobial activities of EOs have been found effective against various microorganisms using direct-contact antimicrobial assays (diffusion or dilution methods) [13, 14]. The oxygenated terpenoids of EOs (alcohols and phenolic terpenes) possess most of the antimicrobial activities, while some hydrocarbons also exhibit antimicrobial effects [15, 16]. Notably, essential oils are volatile, natural aromatic compounds, having distinct fragrance, and are extracted by distillation method from their parts (leaves, stems, bark seeds, fruits, roots, and plant exudate). Various methods have been applied for extraction of essential oils such as water or steam distillation, solvent extraction, extraction under pressure, and supercritical fluid and subcritical water extractions, and other distillation; for example, Clevenger method is the most popular, widely used, and cost effective method for producing most of the essential oils in the world [17]. In this study* T. ammi *and* M. fragrans *were selected for extraction of essential oil, GC-MS analysis, insecticidal activities, and antibacterial activities.

## 2. Materials and Methods

### 2.1. Collection and Preparation of Spices

The fresh materials collected from “spices market” Jaipur, Rajasthan, India, were identified by the Department of Botany, University of Rajasthan, Jaipur, and kept in air tight container at cool and dry place (Table 1).

### 2.2. Extraction Procedure

The essential oils were extracted using 100 gm of sample in 600 mL boiled distilled water placed in round bottom flask of Clevenger's unit, separately. Temperature control burner was set at 70°C to 90°C with adjustment interval of five minutes to fifteen minutes throughout extraction. The volume of essential oils was measured by calibrating tap and separated using decantation method. The separated essential oils were dried over anhydrous Na_2_SO_4_ and kept at −4°C till further use.

## 3. Gas Chromatography of Essential Oil

Gas chromatography analyses of essential oils were done using a Finnigan Focus Gas Chromatograph, Thermo Electron Corporation, with capillary column of SUPELCOWAX (30 mm × 0.25 mm × 0.25 mm) thickness. GC ramp temperature was programmed as follows: initial temperature 80°C held for four minutes with rise of 4°C/min up to 240°C. The carrier gas used was He (helium) at the rate of 10 mL/min at constant volume. The column pressure corresponded to 100 Kpa., injection port temperature was set to 200°C, and detector temperature was 240°C, respectively. Further, oven conditions were programmed as follows: maximum temperature 240°C, prep run time 10 min, equilibration time 0.50 min, and oven run time 49 min. Intel SSL temperature was set at 200°C and split flow was 10 mL/min.

## 4. Determination of Insecticidal Activity of Essential Oil

### 4.1. Insect Cultures

The Indian meal moths culture (*P. interpunctella*) was obtained from the Department of Zoology, University of Rajasthan, Jaipur, and maintained continuously on a feature diet (10% glycerol, 50% dried apricot, and 40% wheat flour with wheat bran mixture). The conditions were maintained at constant temperature (27 ± 1°C), photoperiod (14L : 10D), and relative humidity (60% ± 5) during experiment [18, 19].

### 4.2. Insecticidal Activity

Adult insects, >48 hours old, reared in the Department of Zoology were used for fumigant toxicity. The samples were prepared by affixing 2 × 2 cm of filter papers impregnated with measured dose of EOs (1–10 *μ*L/L air) at upper part of “cotton plugged” Erlenmeyer flask (100 mL). Each sample was inoculated by introducing randomly selected ten adults of* P. interpunctella* and then stored in the incubator under constant conditions (25 ± 1°C, 65 ± 5% RH, and 12L : 12D). Nonimpregnated sample was used as control. The time-frame of 3 to 24 hours was used for calculating insect mortality [20, 21]. The data were analyzed by Abbott's formula [20], SPSS 16, and means were separated at the 5% significance level by the least significant difference test.

#### 4.2.1. Antibacterial Activity of Essential Oil

The dilutions of individual EOs and their combination were prepared by using solvent DMSO (Dimethyl sulfoxide), 1 : 4 for each EO and 1 : 1 : 8 for CEO, where 20 *μ*L was used as experimental volume of each dilution. The* M. fragrans* and* T. ammi* were screened for their antibacterial activity against three Gram (−) bacterial strains (*E. coli*-MTCC 443,* P. vulgaris*-MTCC 1771, and* K. pneumoniae*-MTCC number 7028) and three Gram (+) bacterial strains (*S. aureus*-MTCC 3381,* B. subtilis*-MTCC 10619, and* B. megaterium*-MTCC 2412) by well agar diffusion method [22]. All MHA plates were inoculated by an individual turbid bacterial suspension (~0.5 McFarland standard), maintained at 1.5 × 10^6^ CFU/mL. Further, the different dilutions of individual EOs and CEO were filled in papered 5 mm well on agar pates and incubated at 37°C for 24 hours. Gentamicin (HiMedia, Mumbai) was used as control. Three replicates were kept in each case and average values were calculated. The zones of inhibitions (diameter in mm) were measured and reported. The Minimum Inhibitory Concentration (MIC) method for all test bacterial strains was also determined [23].

## 5. Result and Discussion

### 5.1. Assessments of Yielding Pattern of Essential Oils

**Figure 1:**
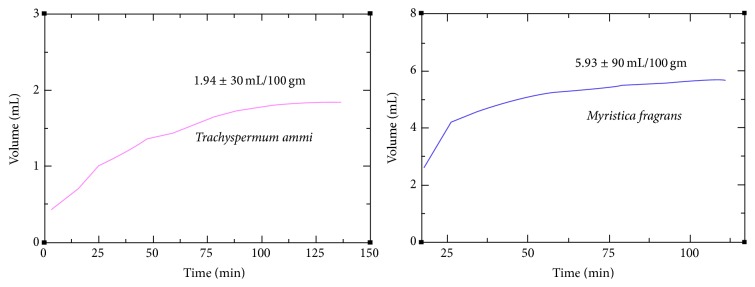
Yielding patterns of essential oils of Indian spices.

In the present study, the EOs of* T. ammi *and* M. fragrans *were extracted by hydrodistillation and their yielding patterns and volume were assessed by observation of the extracted volume after regular intervals (10 min). Evidently, both Indian spices produced essential oil and showed contrastive yielding pattern. The extracted volumes of* T. ammi *and* M. fragrans *were observed to be 1.94 ± 30 mL/100 gm and 5.93 ± 90 mL/100 gm, respectively (Figure 1).

The results showed that initial release of EO of* T. ammi *started within 5 minutes and continuously obtained nearly the same volume until 140 min. In case of* M. fragrans, *the initial release started after 15 minutes whereas maximum yield was obtained within 40 minutes. The release of EO of* M. fragrans *was continued until 125 minutes.

Rabak [24] studied different conditions of* Mentha piperita *L. for obtaining maximum EO yield, that is, maturation time of flowers, alteration in soil, and other climatic conditions, and observed that oil yield may reduce if plants are dried before distillation which may favor the formation of esters and the production of free acids. He also revealed that oil yield in mint plants decreases with maturation which may be due to the increase in the percentage of esters. According to Marotti et al. [25], production and composition of EO were affected by ontogenic stage and pedoclimatic conditions of the plant and it is observed that extended photoperiod is indispensable for plant evolution and yield. The yield of essential oil may vary for spices which depends on species (differing with family), quality (chemotype of the plant), condition (fresh or dry), layout of plant material (e.g., leaf/stem ratio), and method of extraction [26].

#### 5.1.1. GC-MS of* T. ammi* and* M. fragrans*

Hydrodistilled EO of the* M. fragrans* nut and seeds of* T. ammi* were analyzed by Gas Chromatography Mass Spectroscopy. The outcome showed that the major compounds of* T. ammi* were Gamma-Terpinolene, Thymol, and p-Cymene, whereas* M. fragrans *contained beta-pinene, alpha-pinene, alpha-thujen, and p-menth-1-en-4-ol in amount of 20.69%, 15.16%, 12.73%, and 11.03%, respectively. The other minor constituents that were found in EO of* M. fragrans *and* T. ammi *are shown in Table 1. Notably, p-menth-1-en-1-ol, Limonene, p-Cymene, alpha-terpinene, Beta Myrcene, beta-pinene, and alpha-pinene were commonly found in both of the EOs. The major bioactive compound of essential oil of* T. ammi *and* M. fragrans *was Gamma-Terpinolene (53.63%) and beta-pinene (20.69%), respectively (Table 2). Gas Chromatography analysis showed that both of the essential oils have specific constituents that may be responsible for specific properties.

#### 5.1.2. Insecticidal Activity of Essential Oils against the* P. interpunctella *Adults

The mortality values significantly increased depending on the increasing concentration of essential oil when the* P. interpunctella *adults were exposed to caraway (*T. ammi*) and nutmeg (*M. fragrans*) (for caraway: *F* = 232.6; d.f. = 1,6; and *p* < 0.0001; for nutmeg: *F* = 96.98; d.f. = 1,7; and *p* < 0.0001). The mortality values reached 30% and 70% when the adults were exposed to 6 *μ*L/L air concentrations of* T. ammi* and* M. fragrans*, respectively, and all the adults were killed by 14 *μ*L/L air or higher concentration. The mortality effect of* M. fragrans *was lower compared to* T. ammi *oils. LC_50_ values of the essential oils tested were 4.33 *μ*L/L air and 6.65 *μ*L/L air for* T. ammi *and* M. fragrans*, respectively. These values revealed that* T. ammi *essential oil exhibited efficient insecticidal activities against* P. interpunctella *adults compared to* M. fragrans *(Table 3). The nonimpregnated sample did not show mortality of* P. interpunctella *adults. Experiments were performed three times and average values were calculated.

**Figure 2:**
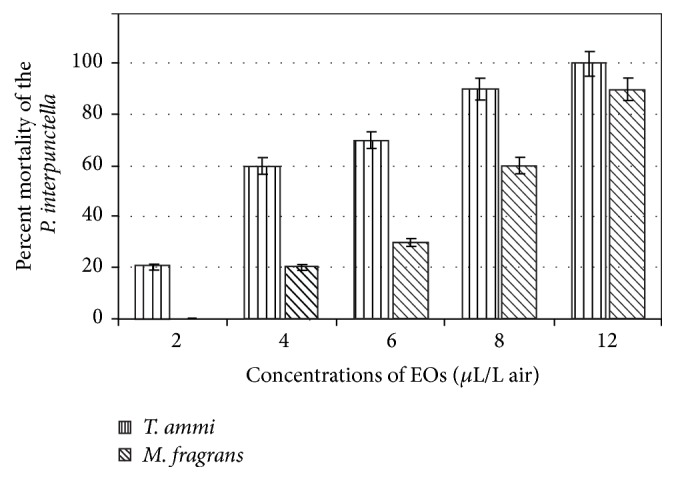
Percent mortality of the* P. interpunctella *after exposure to* M. fragrans *and* T. ammi *essential oils.

The mortality is correlated to EO concentration and exposure time; that is, outcomes (Figure 2) reveal a progressive hike in the mortality rate of* P. interpunctella *with increment in EO concentrations and the exposure time. The same kind of results was also observed by Ahmed (2006) [27]. The divergence obtained among the mortalities was due to the differences in volatilities of both EOs. Huang et al. [28, 29] suggested that most of monoterpenes are highly effective on insects due to their high volatility.

#### 5.1.3. Antibacterial Activity

**Figure 3:**
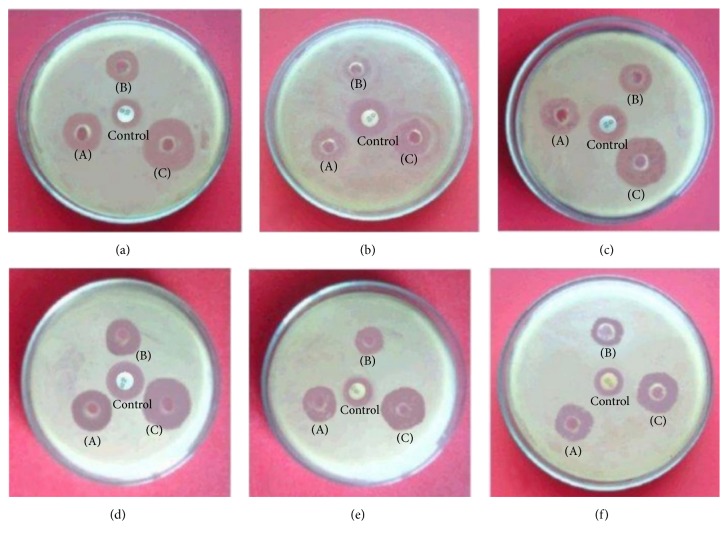
Zone of inhibition. (A)* M. fragrans*, (B)* T. ammi*, and (C) combination of EO against both Gram (+) and Gram (−) bacterial strains, that is, (a)* S. aureus*, (b)* E. coli*, (c)* P. vulgaris*, (d)* B. subtilis*, (e)* K. pneumoniae*, and (f)* B. megaterium*.

**Figure 4:**
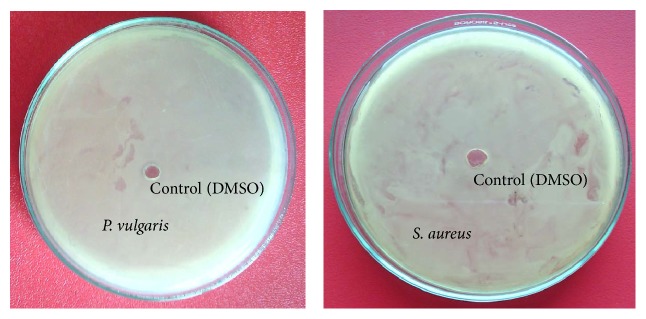
Activity of negative control (DMSO).

The antibacterial activities of dilution of individual EO and CEO of* T. ammi *and* M. fragrans *were evaluated by agar wall diffusion method [30]. The zones of inhibitions (ZOIs) for* T. ammi *were found 18 mm, against* S. aureus, P. vulgaris, B. subtilis, *and* B. megaterium, *and 16 mm for* K. pneumoniae *and 14 mm for* E. coli*. The* M. fragrans *have slightly higher ZOIs compared with* T. ammi *which were found 20 mm against* S. aureus, B. subtilis, *and* B. megaterium *and 18 mm for* K. pneumoniae, P. vulgaris, *and* E. coli.* Notably, ZOIs of mixture of EO were found high as compared with single EO and control (gentamicin) against all test bacterial strains. The highest ZOI was observed in case of CEO against* S. aureus, P. vulgaris, *and* B. subtilis*, that is, 24 mm. These studies indicated that essential oils in combination were more effective against all test bacterial strains at a very low concentration (Figure 3). The antibacterial activity of DMSO (negative control) was evaluated against all six test microorganisms and all microorganisms found resistance for DMSO (Figure 4).

The modified dilution method of Rios [31, 32] was adopted to determine Minimum Inhibitory Concentration (MIC). The dilutions of each of the essential oils and their combinations prepared previously for antibacterial assessment were used as stock solution for delivering assorted concentrations of both EO and CEO, that is, 2 *μ*L, 4 *μ*L, 6 *μ*L, 8 *μ*L, and 10 *μ*L and 12 *μ*L, 14 *μ*L, 16 *μ*L, 18 *μ*L and and 20 *μ*L. The 4 mL nutrient broth containing tubes were treated with each of the experimental concentrations of both EO dilution and CEO followed by inoculation (~0.001 mL, HiMedia, Flexiloop) by using an individual turbid bacterial suspension, maintained at 1.5 × 10^6^ CFU/mL. The tubes were then incubated at 37°C for 24 hours to determine the MIC. The bacterial growth in each sample was determined by observing OD on UV-visible spectrophotometer (Shimadzu UV-1800) at 600 nm after 24 h that represented the Minimum Inhibitory Concentration (MIC). The experiments were carried out at least three times aseptically.

MIC of CEO against* S. aureus, E. coli, P. vulgaris, B. subtilis, K. pneumoniae, *and* B. megaterium *were found 8 *μ*L, 12 *μ*L, 8 *μ*L, 8 *μ*L, 6 *μ*L, and 12 *μ*L, respectively. The dilution of* T. ammi *showed MIC were 8 *μ*L, 12 *μ*L, 10 *μ*L, 10 *μ*L, 8 *μ*L, and 14 *μ*L against* S. aureus, E. coli, P. vulgaris*,* B. subtilis*,* K. pneumoniae,* and* B. megaterium,* respectively. The dilution of* M. fragrans* showed MIC were 10 *μ*L, 14 *μ*L, 12 *μ*L, 12 *μ*L, 10 *μ*L, and 16 *μ*L against* S. aureus*,* E. coli*,* P. vulgaris*,* B. subtilis*,* K. pneumoniae,* and* B. megaterium,* respectively (Table 4).

In the present study synergistic effects of essential oils were studied and CEO exhibited excellent antibacterial activity against all test bacteria, as ZOIs of CEO were more significant as compared to single EO and reference antibiotic. Moreover, MIC determined by modified dilution method for CEO was found at a low concentration compared with MIC of single EO. Present study concords with the result of Gutierrez et al. [33].

## 6. Conclusion

Essential oils are natural plant products containing various components of interest having antimicrobial as well as insecticidal activities. In the present study, it was revealed that both Indian spices have distinct yielding patterns and markedly give EO 1.94 ± 30 mL/100 gm (*T. ammi*) and 5.93 ± 90 mL/100 gm (*M. fragrans*) on hydrodistillation. It is concluded that most of antimicrobial and insecticidal activities of essential oil may be due to oxygenated terpenoids like phenolic terpenes, phenylpropanoids, and so forth. It is also revealed that essential oils have promising potential to inhibit disease causing organism and may be used in contest with agromaterials. The present technique used for extraction of essential oil was found as the promising method for the extraction of essential oil from plants as this process preserves the maximum qualities of plants. Outcomes of the present study showed that both of the essential oils have specific type of constituents that were responsible for effective antibacterial and insecticidal activities against bacterial strains and insects. This study may encourage researchers aiming at possible applications of essential oils and their combination in food, pharmaceuticals, health science, and cosmetology fields.

## Figures and Tables

**Table 1:** Indian spices (*T. ammi *and *M. fragrans*).

Spices	Family	Availability	Part Used
*Trachyspermum ammi*	Apiaceae	Eastern Mediterranean, Egypt, and India	Intact fruit

*Myristica fragrans*	Myristicaceae	Moluccas of Indonesia, Guangdong and Yunnan in China, Taiwan, Malaysia, Grenada in the Caribbean, Kerala in India, Sri Lanka, and South America	Coarsely grinded nuts

**Table 2:** Chemical constituent of the essential oils obtained from *T. ammi* and *M. fragrans*.

Compounds detected	IUPAC name	*M. fragrans* abundance (%)	*T. ammi* abundance (%)
Alpha-thujen	4-Methyl-1-propan-2-ylbicyclo[3.1.0]hex-3-ene	12.73	—
Alpha-pinene	4,6,6-Trimethylbicyclo[3.1.1]hept-3-ene	15.16	2.91
Camphene	(1S,4R)-3,3-Dimethyl-2-methylidenebicyclo[2.2.1]heptane	4.92	—
Beta-pinene	6,6-Dimethyl-4-methylidenebicyclo[3.1.1]heptane	20.69	8.95
Beta Myrcene	7-Methyl-3-methylideneocta-1,6-diene	4.11	1.11
Alpha-phellandrene	2-Methyl-5-propan-2-ylcyclohexa-1,3-diene	3.91	—
3-Carene	4,7,7-Trimethylbicyclo[4.1.0]hept-3-ene	2.84	—
Alpha-terpinene	1-Methyl-4-propan-2-ylcyclohexa-1,3-diene	4.32	2.62
p-Cymene	1-Methyl-4-propan-2-ylbenzene	7.81	13.50
Limonene	1-Methyl-4-prop-1-en-2-ylcyclohexene	8.06	0.57
Beta-ocimene	(3E)-3,7-Dimethylocta-1,3,6-triene	4.04	—
Gamma-Terpinolene	1-Methyl-4-propan-2-ylidenecyclohexan-1-ol	5.15	53.63
Alpha-Terpinolene	1-Methyl-4-propan-2-ylcyclohexa-1,3-diene	4.31	—
Linalool	3,7-Dimethylocta-1,6-dien-3-ol	6.91	—
p-Menth-2-en-1-ol	1-Methyl-4-propan-2-ylcyclohex-2-en-1-ol	3.30	—
p-Menth-1-en-1-ol	(1R)-4-Methyl-1-propan-2-ylcyclohex-3-en-1-ol	11.03	0.39
Alpha-terpineol	2-(4-Methylcyclohex-3-en-1-yl)propan-2-ol	6.72	—
Eugenol	4-Allylcatechol 2-methyl ether	7.12	—
Myristicin	4-Methoxy-6-prop-2-enyl-1,3-benzodioxole	6.44	—
Elemicin	1,2,3-Trimethoxy-5-prop-2-enylbenzene	8.81	—
Sabinene	4-Methylidene-1-propan-2-ylbicyclo[3.1.0]hexane	—	0.44
Beta-phellandrene	3-Methylidene-6-propan-2-ylcyclohexene	—	0.91
Cis-beta-terpineol	cis-4-Isopropenyl-1-methylcyclohexanol	—	0.39
Thymol	5-Methyl-2-propan-2-ylphenol	—	16.77

**Table 3:** LC_50_ values of *M. fragrans *and *T. ammi *essential oils against the adults of *P. interpunctella.*

EO	LC_50_ (95% CL) (*μ*L/L air)	*χ* ^2^ (d.f.)	Slope
*T. ammi *	4.33	21.32 (7)	1.207 ± 0.07913
*M. fragrans *	6.65	23.61 (8)	0.7839 ± 0.05043

**Table 4:** Antibacterial activity of CEO, *M. fragrans,* and *T. ammi*.

Bacterial sp	Dilution of EO		
	CEO *µ*L (12.5% v/v)	*M. fragrans µ*L (25% v/v)	*T. ammi µ*L (25% v/v)
*S. aureus*	8	8	10
*E. coli*	12	12	14
*P. vulgaris*	8	10	12
*B. subtilis*	8	10	12
*K. pneumoniae*	6	8	10
*B. megaterium*	12	14	16
